# Proceedings of the Organon Society of Boston

**Published:** 1888-04

**Authors:** 


### PROCEEDINGS OF THE ORGANON SOCIETY OF BOSTON.

### REGULAR MEETING OF ORGANON SOCIETY, BOSTON, FEB. 9TH, 1888.

The introduction to the *Organon* was taken up, and reading began at middle of thirty-seventh page.

In regard to the remark that Cinchona was specific as a homœopathic remedy only in genuine swamp-ague, provided it is used in the absence of Psora, Dr. Tompkins inquired if it was not too sweeping a statement to say that Cinchona or any remedy was a specific for a disease.

Dr. Wesselhœft said that Hahnemann always said it was wrong to treat the disease, and especially wrong to treat such a disease as chills and fever, with a single remedy, Cinchona, when the disease varies so much. This was probably only a passing remark, like that in regard to Merc. in pure syphilis. Given a case of pure syphilis, or a case of pure swamp-ague without psoric symptoms, Merc. or China might be indicated, and, if so, they would cure, but they certainly should not be given unless they were indicated by the symptoms.

Dr. Wesselhœft also spoke of the case of Ægidi, the body physician of a prince of Russia and a student of Hahnemann. The Prince and his suite were traveling, and several of them were attacked with chills and fever. Ægidi cured them all but one, a Saxon officer; with him he could do nothing. In his despair he wrote to Hahnemann, saying that nothing seemed to help. Hahnemann replied that probably there had been a suppression of a former ague, and, as the patient was a native of Saxony, it was not at all unlikely that he had had the ague that was epidemic there some years before, in which Cantharis was the epidemic remedy, on account of the urinary symptoms which were present in almost every case. Now if the patient had the ague at that time and it had been suppressed, Cantharis was the remedy.

Upon inquiry, Ægidi found that such was the case; his patient at that time, some years before, was in Saxony; had the ague that was then prevalent, and it had been suppressed with Cinchona. Ægidi therefore gave him Cantharis, as Hahnemann recommended, and he quickly recovered.

Dr. Bell said he would like to ask the opinion of those present in regard to the Quinine habit. He said he had found it a very common thing for people to take Quinine for everything, a little cold, loss of appetite, etc. He had found that neuralgias and anæmia following the use of Quinine were very hard to cure.

Even St. John Roosa, the aurist, had warned his allopathic colleagues of the danger of deafness from their indiscriminate use of Quinine.

Dr. Cobb spoke of an allopathic physician who took his Quinine regularly, morning, noon, and night, and also a lady who took it in much the same manner, and they both looked as if they were suffering from Quinine poisoning, as they probably were.

Dr. Wesselhœft said he once heard Hering say, at the time when spiritualism was so widespread, that when the statistics of insanity were known, it would be found that Cinchona and not spiritualism was the cause of so much insanity in America. Hering also said that when he smelt Carbolic acid in a house with scarlet fever, he was always afraid of a death twenty years later.

Bilroth, of Vienna, came out with his cases of Carbolic acid poisoning, and Hering's prediction as to Quinine may prove true at some future date. The amount of Quinine consumed yearly in America is stupendous.

Dr. Wesselhœft also spoke of a case his father treated, a relative who had lived in Central America and had had Chagres fever. He came to Boston to make a visit, and about lived on Quinine. He was a very leathery looking man. Soon after his arrival here, he was taken ill with chills and fever. His Quinine did not help him, and he sent for the elder Dr. Wesselhœft. It took a long time to cure him, and he had two chills daily for six weeks. He finally recovered and returned to Central America. Some time after he wrote to Dr. Wesselhœft to tell him how exceedingly well he had been, and, strange to say, he had not had a chill since his return; he had never been so long without one since living in Central America.

Another case was that of a lady in Jamaica Plain, who had been taking Quinine for some time for chills contracted at West Point, N. Y. They would return every time she visited West Point. She was treated homœopathically, and the next year she went to West Point and did not have a chill, nor has she had any since, and she has been there several times.

Dr. Wesselhœft also said he had now a case of Quinine cachexia of twenty years' standing, with sycosis of fifteen years, and a fresh sycosis of two years' standing. He has been under very poor homœopathic treatment for two years.

The first thing, after being under Dr. W.'s treatment, was a return of the chill, which he had for nine days, until Euphorbium was given, which controlled it. After the chills began to get better, gonorrhœal symptoms came on and the urinary symptoms were very severe. This was *formerly* treated by injections. Now he feels very much better generally, although the urinary symptoms cause a great deal of suffering, and Dr. W. said he should not give a new remedy. The only way to unravel these things is to let the remedy act.

Now, when a case is generally getting better he dreads to interfere with a new remedy.

He also spoke of a case of pericarditis following rheumatism, which he saw in consultation, and which had had the following remedies, selected with much care: Bry., Kalmia, Spig., and Lach.

It began in shoulders and went down; the joints were not very red. Bry. was given first.

When the limbs began to improve the heart was affected and Kalmia was given, and the pains went back to the limbs again. Nothing was given. Soon there was another attack on the heart, which was relieved by Spig., and was followed by general improvement for some days. Then the heart began again to be affected. The patient would wake from sleep confused. There was profuse perspiration without relief. Most of the pains were in the region of the left lower ribs in front; the symptoms were all left-sided. Then Lach.$^{cm}$ was given and Dr. W. sent for in consultation. When he got there he found the pains had shifted to the right lower rib region and the heart's action was perfectly regular. The attending physician said that three or four hours before when Lach. was given the heart-sounds were so jumbled up that they could not be distinguished and the action was very irregular and intermittent. It was a beautiful result, and the patient made a good recovery.

In regard to Hahnemann's remarks on Digitalis, page 39, Dr. Wesselhœft said that he recalled the case of a young lady who had rheumatism with heart complications. When the joints were worse the heart was better. The indicated remedy cured her and she was well for two years. Then she moved to New York, and there she had another attack. An allopathic physician was called, Salicylic acid was given, and the joints grew less painful but the heart became affected. Then Digitalis was exhibited, and on the third day she had a very marked Digitalis symptom, "great sinking at the stomach." The attending physician said it was nothing and told them to feed her. The next day there was more sinking and the next day she was dead.

Dr. W. said he did not believe there was an allopathic physician who knew that Digitalis produced great sinking at the stomach. He said it was the rarest thing to cure a patient poisoned with Digitalis, but he thought he had cured one lately. He read from Bœnninghausen that the homœopathist must have very little hope of affecting a cure after Digitalis.

Dr. Wesselhœft spoke of the fact that physicians *make* heart diseases, by saying that the pulse must be reduced and giving Digitalis, as they say that the bowels must be moved, or the temperature reduced. Homœopathists have no business to base a prescription upon the condition of the pulse, temperature, or bowels. They should be watched, but never prescribed for alone; they may aid in the prognosis, but in regard to indicating a remedy they have nothing to do with it.

Dr. Bell said that people should be told that such things as Digitalis and Quinine are harmful, and physicians should be more outspoken about them. He thought the condition of the pulse and temperature might tell us of the favorable action of a remedy.

Dr. Cobb related a case of an old man seventy years of age. In the previous year there was no sign of heart disease, but after awhile hypertrophy developed, and the family becoming alarmed, they wished a so-called homœopathist to be called in consultation. He came and advised Digitalis in heroic doses, that is, thirty drops of the first decimal solution in a tumbler of water, a tablespoonful to be taken every hour for three days.

At the end of that time, the patient being no better, he wished to give the tincture; he said the man would die anyway in three days and the tincture would relieve him and make him feel easier. Dr. Cobb protested and the consulting physician withdrew. The patient lived three months and then his heart stopped. Dr. C. thought he would have lived much longer if the Digitalis had not been given at all.

Dr. Kennedy spoke of a case concerning which a young homœopath consulted him, a case of heart disease. Dr. K. advised Puls., and the young man said he had thought of that but could not find that Puls. had any pathological action on the heart. He gave it, however, and the patient improved, but the young man was much worried that the pulse and temperature kept up.

Dr. Wesselhœft said it reminded him of what Hufeland once said of Hahnemann, "that he relieved the symptoms, but what did he do with the disease?" Hahnemann ridiculed him unmercifully for considering the disease an entity.

Dr. Tompkins spoke of a case of heart disease with dropsy under the care of a so-called homœopathic physician. The patient was getting worse all the time and a member of the family went on to consult Guernsey. He gave him two powders of Puls.$^{2m.}$ and told him to give the patient one dose, and in thirty-six hours if no better to give the other. After the first dose the patient began to improve and got along all right. The attending physician learned nothing from this, however; he thought the third would have done just as well, but he had not given it.

Dr. Wesselhœft spoke of a Chamomilla case he had when a young man. He was called to see a child which was troubled with a lot of wind in its bowels; wanted to be carried all the time; greenish diarrhœa, etc.; a very evident Cham. case. While preparing the remedy he asked if the child had been taking anything, and found they had been giving it Cham. in a very low potency every three hours for a week. He had a Cham. proving instead of a case.

Dr. Hastings asked how it happens that the allopaths do sometimes seemingly help patients that have been under good homœopathic treatment.

Dr. Wesselhœft said that such things are usually suppressions where the troublesome symptoms disappear for awhile to return again, or they are really homœopathic cures. He had lately treated a case very carefully, a young lady with anæmia and tape-worm. She did not improve and went to an allopathic physician. He gave her Iron with evidently very beneficial results. Now there seemed to be no indication for Iron whatever, and time would show whether it was a homœopathic cure or an allopathic suppression.

Dr. Bell spoke of a case of a child with a chronic cough in which Opium and cough mixtures had been given for a long time. Because the child was not cured entirely in two or three days, they went back to the mixture again and after awhile the cough stopped.

*Per contra*, Dr. Wesselhœft spoke of a case where opiates and cough mixtures were given for months without benefit and the cough had to be cured homœopathically.

Adjourned to February 23d.

---

### Regular Meeting of Organon Society, Feb. 23d.

Dr. Bell was absent in New York and Dr. Wesselhœft was out most of the evening.

Dr. Nichols continued the reading of the introduction to the *Organon.*

Dr. Jameson spoke of a case of a woman who was nearly dead from repeated hemorrhages. She had with it all watery, colorless stools with cramps. Verat. alb., high, at once relieved.

Dr. Nicols related a case of epistaxis cured by Millefol.$^{cm}$ It was of years standing and was relieved at once, though various remedies and expedients had been used, including compression and plugging.

He also spoke of the case of a man nearly dead from the abuse of Digitalis, who was cured by Colch.

The remedy was suggested by his *disgust for food.*

After finishing the introduction Dr. Nichols read from Stratton's translation examples of homœopathic cures performed unintentionally by allopathic physicians.

Dr. Cobb gave an instance of homœopathic prophylaxis before she studied Homœopathy, where she directed the leaves of the Rhus to be macerated and then placed in water. This was to be taken internally, and in this way a whole neighborhood was protected from the poisoning.

Another instance was related where numbers who had been affected by Rhus were relieved by a high potency of Rhus. The question was asked, was this isopathy? In the discussion which followed it was shown that the mucous membrane was not at all affected; the drug, the leaves of the Rhus, could be chewed with impunity.

Dr. Wesselhœft thought that persons once poisoned were more likely to have a recurrence when exposed, and quoted a number of cases as examples.

He also spoke of persons being poisoned by ferns which grew in close proximity to the Rhus. These ferns poison people after they have been gathered and are being used for decorating or some such purpose. This may explain numerous cases where the patients are sure they have handled no Rhus leaves or twigs, but they have been assisting in some decorating work with ferns, etc. It such cases it would be interesting to know where the ferns came from.

Dr. Wesselhœft related two cases of erysipelas. One, a facial erysipelas, began on the right side and went to the left, with formation of blisters, and extended over the whole face. The chief aggravation was after midnight; the upper part of the body, face, and chest was very hot, lower part cool. $\text{Rhus}^{\text{cm}}$ was given, and later $\text{Rhus}^{\text{cmm}}$. The inflammation extended, the blisters changed to yellow honeycombed crusts, extreme lassitude came on, the patient, a stout blonde, inclined to sadness, became much depressed, wished to die. $\text{Graph.}^{\text{cm}}$ was given and was followed by marked relief.

The other case was also facial, the eruption was smooth, the patient was very delirious, with constant talking and wild, staring eyes. $\text{Stram.}^{\text{cm}}$ cured.

The question of the efficacy of Bell. as a prophylactic in scarlet fever was raised, and instances were quoted by Dr. Nichols which seemed to show that it does afford protection from the disease. Other instances were given where Bell. was not given and only one member of the family was affected.

Dr. Hastings related a curious case in which the eruption began on the legs and progressed upward and afterward the rash appeared on the face and went downward in the usual manner.

Cases were referred to where eruption first appeared on the back.

Adjourned to March 8th. S. A. Kimball, *Secretary*.

